# Dietary phytochemical indole-3-carbinol regulates metabolic reprogramming in mouse prostate tissue

**DOI:** 10.1007/s11095-025-03820-8

**Published:** 2025-02-04

**Authors:** Rebecca Mary Peter, Md. Shahid Sarwar, Lujing Wang, Pochung Chou, Chao Wang, Yujue Wang, Xiaoyang Su, Ah-Ng Kong

**Affiliations:** 1 Department of Pharmaceutics, Ernest Mario School of Pharmacy, The State University of New Jersey, RutgersPiscataway, NJ 08854 USA; 2 Graduate Program of Pharmaceutical Sciences, Ernest Mario School of Pharmacy, The State University of New Jersey, RutgersPiscataway, NJ 08854 USA; 3 Metabolomics Shared Resource, Rutgers Cancer Institute of New Jersey, New Brunswick, NJ 08901 USA; 4 Department of Medicine, Rutgers-Robert Wood Johnson Medical School, New Brunswick, NJ 08901 USA

**Keywords:** Indole-3-carbinol, Nrf2, prostate cancer, Pten, untargeted metabolomics

## Abstract

**Purpose:**

Indole-3-carbinol (I3C) is shown to possess multiple pharmacological activities such as anti-inflammatory, antimicrobial, antioxidant, antiviral, and anti-cancer activities. It is widely accepted as modulator of multiple signaling pathways particularly those related to cell cycle, cell growth and division, angiogenesis, apoptosis and immunity. We explored the metabolic reprogramming based on treatment with I3C in mice prostate tissue.

**Methods:**

In this study we utilized Pten knockout (KO)-induced prostate tumorigenesis mouse model to examine mechanism of action of I3C via metabolic rewiring. Phosphatase and tensin homolog deleted on chromosome 10 (Pten), a tumor suppressor gene is frequently found to be mutated or deleted in prostate cancer. Untargeted metabolomics was performed using liquid-chromatography mass-spectrometry (LC–MS) based platform to investigate Pten-dependent and Pten-independent metabolic targets of I3C.

**Results:**

The most impacted pathways by I3C included pyrimidine metabolism, arginine and proline metabolism, porphyrin metabolism, citrate cycle and lipoic acid metabolism.

**Conclusion:**

These pathways taken together help in understanding the overall health beneficial effects of I3C.

**Supplementary Information:**

The online version contains supplementary material available at 10.1007/s11095-025-03820-8.

## Introduction

Prostate cancer (PCa) is a leading cause of male cancer deaths in the developed world [1]. Prostate carcinogenesis is a resultant of chronic inflammation due to consumption of high dietary fats and heterocyclic amines or due to unknown pathogenic infections [2]. Because of the late stage diagnosis of the disease, surgical methods seem to be the only treatment options. The phosphatase and tensin homolog deleted on chromosome 10 (Pten) is a tumor suppressor gene and is frequently mutated or deleted in various human cancers [3, 4]. Disorders such as Cowden syndrome and related diseases have been associated with germline mutation in the Pten gene. [5]. Pten mutation is reported as the most common genetic alteration in 30% of primary prostate cancer cases and 63% of metastatic prostate cancer cases [6].

Cellular energy metabolism consists of a series of biochemical processes including glycolysis, oxidative phosphorylation via the tricarboxylic acid (TCA) cycle, lipogenesis and urea cycle. These pathways typically involve specific nutrients including amino acids, fatty acids and carbohydrates [7]. Metabolic reprogramming is an important hallmark of PCa development and progression. Tumor-specific metabolic alterations provide great potential to stratify patient’s risk and identify new biomarkers. Co-targeting central metabolic nodes instead of targeting a single gene or pathway can provide useful information about the genetic events or activated pathways to improve cancer-specific therapeutic efficacy. Therefore, combination of drugs targeting prostate cancer metabolism and standard therapies like chemotherapy and targeted therapy approaches may prove beneficial.

Indole-3-carbinol (I3C) is a dietary phytochemical belonging to the *Brassicaceae* family, which is extensively studied as a regulator of various signaling pathways and targets that mediate cell division, angiogenesis or apoptosis [8, 9]. Preclinical and clinical studies suggest that I3C has great potential in preventing chronic diseases including cardiovascular disease, obesity and diabetes and [8]. Previously, in our lab we established antioxidant and anti-inflammatory mechanism of I3C based on regulation of glutathione S-transferase Ya subunit gene at transcription level and regulation of phase II drug metabolizing and antioxidant genes mediated by Nrf2 [10–15]. I3C is also identified as a promising chemotherapeutic agent against gastrointestinal tumors [16]. Based on treatment with I3C in *in-vitro* studies conducted in prostate cancer cells, I3C was revealed as an up regulator of phase I and phase II detoxification enzymes, inducer of apoptosis via G1 cell-cycle arrest and inhibitor of Ak strain transforming (Akt) and nuclear factor kappa B (NF-κB) which are well known molecular targets of cancer therapy [17].

**Fig. 1:**
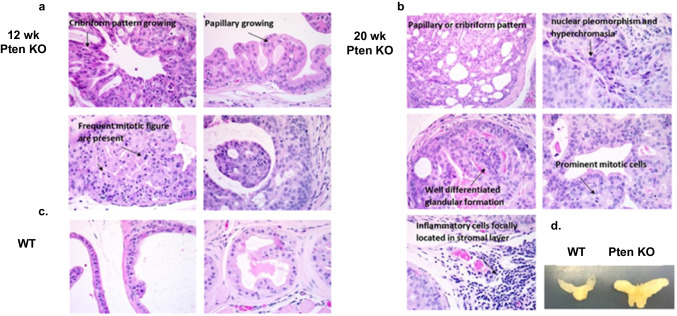
Histopathological evaluation of prostate-specific PTEN − / − (KO) mouse prostatic adenocarcinoma model previously established and published in our lab [19] (**a**) 12-week -old PTEN KO mice with PIN progression lesions (**b**) 20-week-old PTEN KO mice with PIN progression lesions (**c**) Wild type mice (**d**) Photograph of prostate tissues obtained from PTEN KO and WT mice

To assess the significance of Pten homozygous deletion in prostate cancer progression, we generated prostate-specific Pten deletion Ptenloxp/loxp;PB-Cre4 mouse model. Our previous studies showed that loss of Pten heterozygosity led to a significant delay in latency of PIN formation and resulted in progression of prostate cancer to metastatic stage [18, 19] (Fig. 1). This essentially mimicks disease progression seen in humans. After establishing this animal model, there is great interest to investigate the effect of I3C on cancer interception via metabolic reprogramming. In this study, we determined metabolic alterations due to I3C diet in prostate specific Pten KO mouse model.

## Materials and Methods

### Chemicals and animal diet

I3C was purchased and blended in AIN-93 M rodent diet (Research Diet, Inc. New Brunswick, NJ, USA) at a final concentration of 1% (w/w). The diet storage temperature was 4°C during the experimentation period. All mice were caged under standard conditions of 12-h light/12-h dark cycle. Water and diet were provided ad *libitum* in accordance with the protocol approved by the Institutional Animal Care and Use Committee (IACUC) at Rutgers University.

### Animal model

**Fig. 2:**
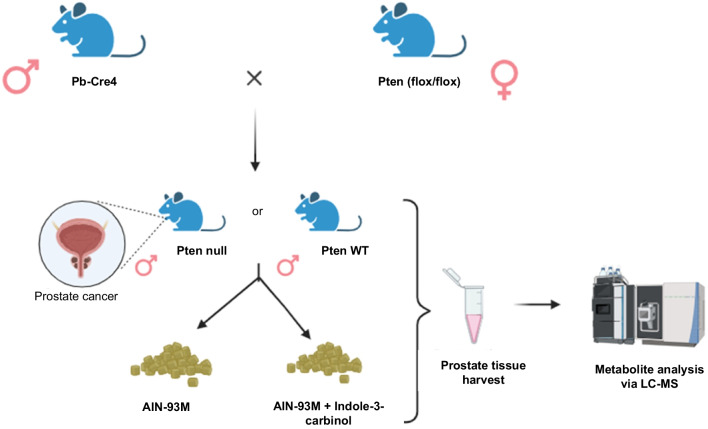
Breeding scheme and experimental design

Pb-Cre4 mice (strain: B6.Cg-Tg(Pbsn-cre)4Prb/Nci) and Pten(flox/flox) mice (C;129S4-Ptentm1Hwu/J) were obtained from the National Cancer Institute, USA and Jackson Laboratories respectively. Prostate-specific Pten knockout (KO) male offspring (Pb-cre/Pten(flox/flox)) were generated at F2 generation by crossing female Pten(flox/flox) mice with male Pb-Cre4 mice. For simplicity, Pb-cre/Pten (flox/flox) mice are referred as Pten-KO, and Pten (flox/flox) mice are referred as Pten wild-type (WT) (Fig. 2). The mice were genotyped appropriately using polymerase chain reaction (PCR), and only the male mice that were cre carriers and homozygous Pten flox/flox were used for the treatment groups. The expected band size were 393-bp for Pb-Cre4 mice (primers: 5’-CTGAAGAATGGGACAGGCATT-3’ and 5’-CATCACTCGTTGCATCGACC-3’), 328-bp for mutant allele of Pten (primers: 5’-CAAGCACTCTGCGAACTGAG-3’ and 5’-AAGTTTTTGAAGGCAAGATGC-3’), 156-bp and 328-bp for heterozygous Pten (primers: 5’-CAAGCACTCTGCGAACTGAG-3’ and 5’-AAGTTTTTGAAGGCAAGATGC-3’).

### Animal grouping

Mice were randomly divided into four experimental groups: Pten WT and Pten KO mice fed on control diet or I3C diet. Mice were sacrificed by CO_2_ asphyxiation at 20 weeks. Mice prostates were harvested immediately and either dissected into ventral & lateral prostate (VLP) or dorsal & lateral prostate (DLP), snap-frozen in liquid nitrogen and stored at − 80°C for downstream analysis. For histopathological evaluation prostate tissues were fixed in 10% phosphate-buffered formalin at room temperature for 24 h.

### *Prostate tissue metabolite analysis *via* LC–MS*

Prostate tissues from the aforementioned four experimental groups were harvested, utilized for organic extraction of cellular metabolites and subjected to liquid chromatography-mass spectrometry (LC–MS) analysis based on the protocol from our previously published paper [18]. Briefly, about 30 mg tissue was pulverized with Yttria Grinding ball using CryoMill at 20 Hz for 2 min to ensure complete homogenization of tissue. Tissue metabolite extraction was performed on ice. Metabolic quenching was done by adding low temperature methanol, acetonitrile and water in the ratio 40:40:20 with 0.5% formic acid. After 20 min incubation, samples were centrifuged at 14,000 g for 10 min. The quenching steps were repeated. Ammonium bicarbonate (15%) was then added to the final supernatant for LC–MS analysis. Methodology for LC–MS is described previously [18]. The metabolite data for each animal group was analyzed using MetaboAnalyst 5.0 software.

### Statistical analysis of individual metabolite ions

Identified metabolites of the aforementioned four experimental groups were analyzed for statistical significance using Two-way ANOVA in GraphPad Prism 9.0.2, considering Tukey test based multiple comparison. Adjusted P-values < 0.05 comparing (i) Pten WT (control diet) and Pten KO (control diet) (ii) Pten KO (control diet) and Pten KO (I3C diet) (iii) Pten KO (I3C diet) and Pten WT (I3C diet) are represented in appropriate figures for the purpose of understanding effect of I3C on mice prostate tissue.

## Results

### I3C regulates Pten dependent metabolic pathways in mice prostate

**Fig. 3:**
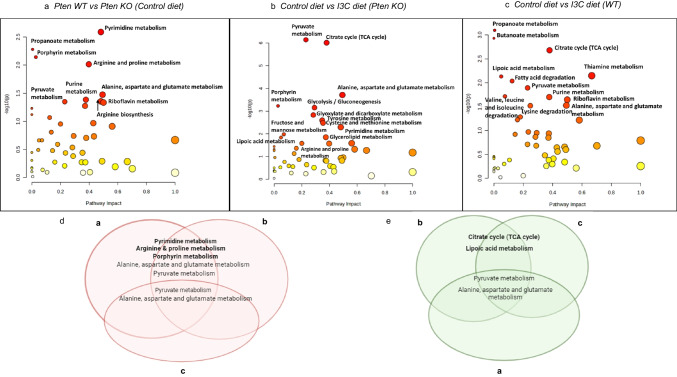
Untargeted metabolic pathway analysis of animal prostate tissues based on different experimental groups (**a**) Most regulated metabolic pathways based on comparison between Pten KO (*n* = 5) and Pten WT (*n* = 5), both fed with control diet. (**b**) Most regulated metabolic pathways based on comparison between control diet (*n* = 5) and I3C diet (*n* = 6) fed to Pten KO mice (**c**) Most regulated metabolic pathways based on comparison between control diet (*n* = 5) and I3C diet (*n* = 3) fed to WT mice. Venn diagram based on (**a**), (**b**) and (**c**) identifies (**d**) most regulated Pten dependent metabolic pathways and (**e**) most regulated Pten independent metabolic pathways targeted by I3C diet in Pten KO mouse model

Based on LC–MS based untargeted metabolomics, we identified 218 metabolites that were analyzed and interpreted to determine the most regulated biochemical pathways. We performed metabolic pathway analysis (integrating pathway enrichment analysis and pathway topology analysis) for two groups data (MetaboAnalyst 5.0) with sample sizes ranging from 3 to 6. Statistical p-values were adjusted for multiple testings. To determine Pten dependent metabolic pathways regulated by I3C, we first identified most regulated metabolic pathways caused by genetic KO of Pten in mice (fed on AIN-93 M control diet) (Fig. 3a). Let it be “x”. We next compared metabolites in prostate tissues of Pten KO mice (fed on AIN-93 M I3C diet) with Pten KO mice (fed on AIN-93 M control diet) to determine significantly regulated metabolic pathways based on difference in diet (Fig. 3b). Let it be “y”. Furthermore, we compared metabolites in prostate tissues of Pten WT mice (fed on AIN-93 M I3C diet) with Pten WT mice (fed on AIN-93 M control diet) **(**Fig. 3c**)**. Let it be “z”. Intersection of Figs. 3a and 3b but excluding Fig. 3c provided the Pten dependent metabolic pathways which were exclusively targeted by I3C diet (Fig. 3d). Alternatively,

$${\varvec{P}}{\varvec{t}}{\varvec{e}}{\varvec{n}}\;\boldsymbol{ }{\varvec{d}}{\varvec{e}}{\varvec{p}}{\varvec{e}}{\varvec{n}}{\varvec{d}}{\varvec{e}}{\varvec{n}}{\varvec{t}}\;\boldsymbol{ }{\varvec{I}}3{\varvec{C}}\;\boldsymbol{ }{\varvec{t}}{\varvec{a}}{\varvec{r}}{\varvec{g}}{\varvec{e}}{\varvec{t}}{\varvec{e}}{\varvec{d}}\;\boldsymbol{ }{\varvec{m}}{\varvec{e}}{\varvec{t}}{\varvec{a}}{\varvec{b}}{\varvec{o}}{\varvec{l}}{\varvec{i}}{\varvec{c}}\;\boldsymbol{ }{\varvec{p}}{\varvec{a}}{\varvec{t}}{\varvec{h}}{\varvec{w}}{\varvec{a}}{\varvec{y}}{\varvec{s}}={\varvec{x}}\cap {\varvec{y}}-{\varvec{z}}$$

Based on this data analysis, we determined that I3C diet significantly modulated pyrimidine metabolism, arginine and proline metabolism and porphyrin metabolism. The list of all identified metabolic pathways along with their statistical significance is provided in supplementary information.

#### I3C targets deregulated pyrimidine metabolism in Pten KO mice prostate

**Fig. 4:**
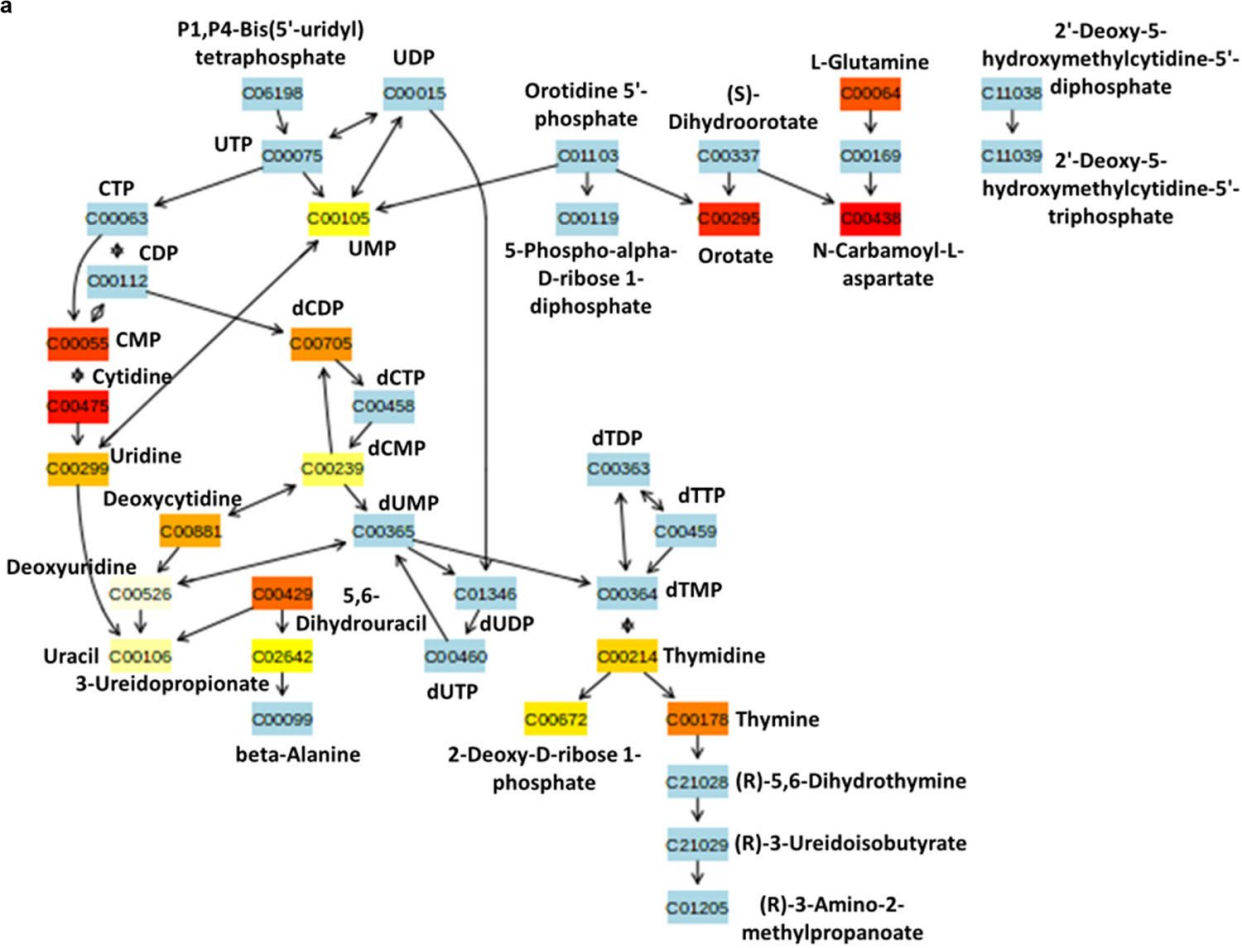

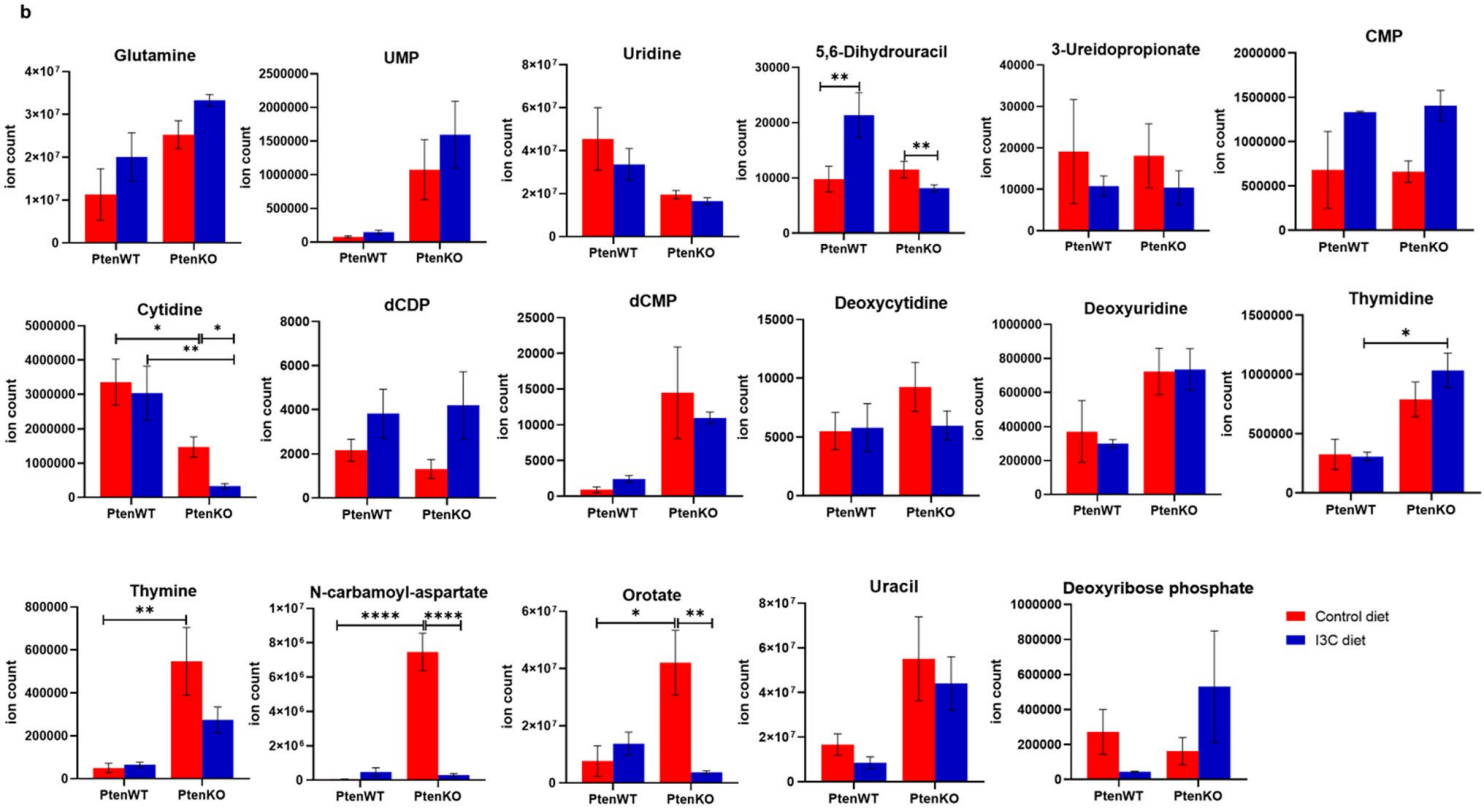
Regulation of Pten-dependent pyrimidine metabolism by I3C in prostate of Pten KO mice (**a**) Metabolite enrichment analysis based on I3C diet in Pten KO mice. Color coded compounds within the pathway: light blue represents metabolites that are not in the data and are used as background for enrichment analysis; other colors (varying from yellow to red) represent the metabolites that are in the data with different levels of significance. (**b**) Expression of pyrimidine metabolism metabolites in prostate tissues of different experimental groups is shown as mean ± SEM. *adjusted *p* < 0.05, **adjusted *p* < 0.01

Pyrimidine metabolism is one of the top Pten regulated metabolic pathways based on prostate tissue metabolite analysis. This is also confirmed in prostate cancer cell lines by Loh *et al*. [20]. We identified that I3C diet significantly targets the perturbed pyrimidine metabolism caused by Pten KO. N-carbamoyl-aspartate, a key intermediate of pyrimidine metabolism was increased by ~ 127 fold with the silencing of Pten, but interestingly I3C substantially reversed this increased effect by downregulating N-carbamoyl-aspartate levels by ~ 22 fold. I3C diet also reduced significantly the overexpression of orotate (5.46-fold elevation) in Pten KO prostate tissue relative to Pten WT, by about 11-fold (relative to untreated Pten KO). In addition, ribonucleoside cytidine showed considerable decrease in Pten KO mice with or without I3C treatment but statistically significant change in its corresponding nucleoside 5′-monophosphate i.e. cytidine monophosphate (CMP) could not be seen (Figs. 4a and b).

#### I3C targets deregulated arginine and proline metabolism in Pten KO mice prostate

**Fig. 5:**
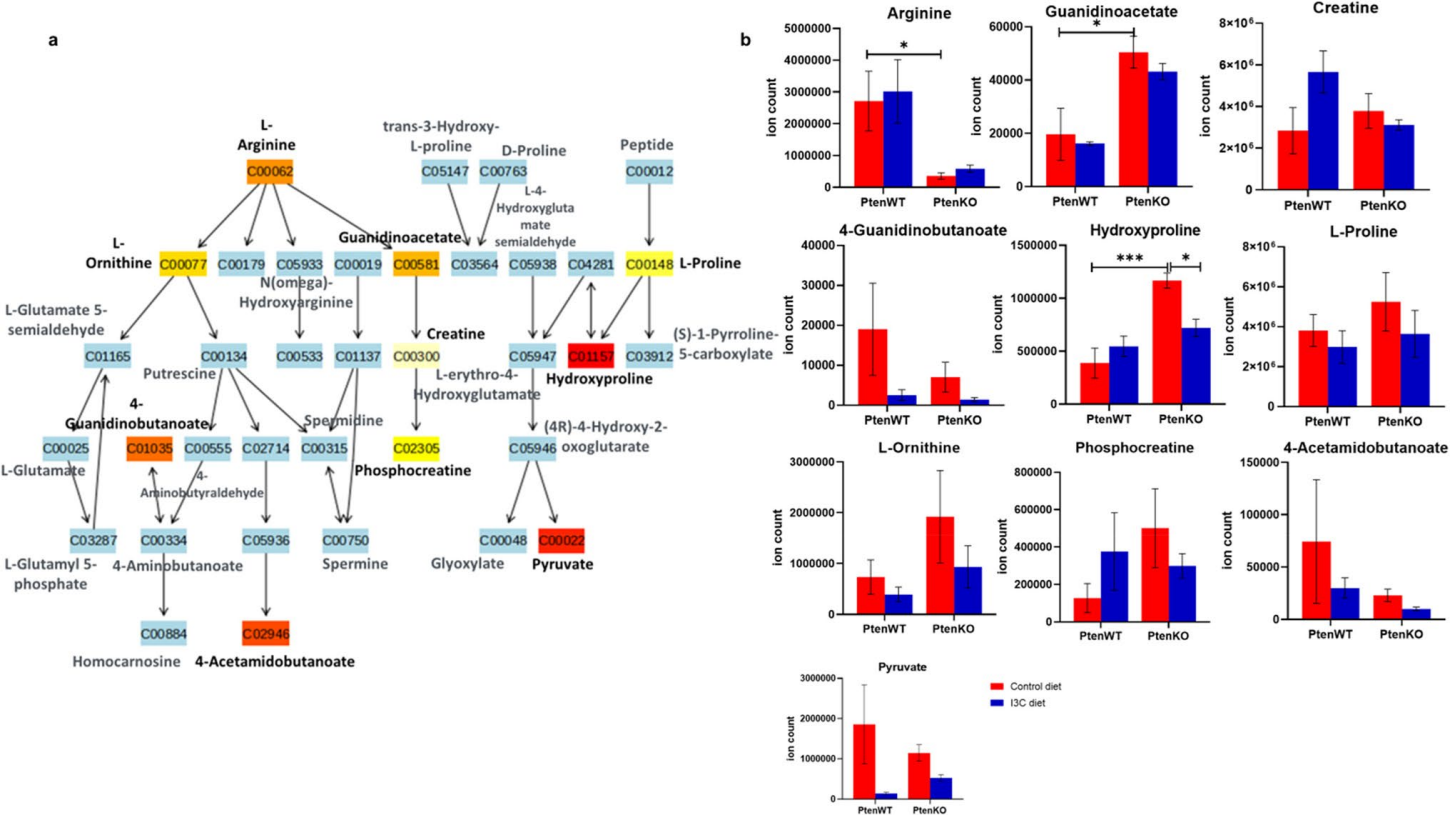
Regulation of Pten-dependent arginine and proline metabolism by I3C in prostate of Pten KO mice. (**a**) Metabolite enrichment analysis based on I3C diet in Pten KO mice. Color coded compounds within the pathway: light blue represents those metabolites that are not in the data and are used as background for enrichment analysis; other colors (varying from yellow to red) represents the metabolites that are in the data with different levels of significance. (**b**) Expression of arginine and proline metabolism metabolites in prostate tissues of different experimental groups is shown as mean ± SEM. *adjusted *p* < 0.05, **adjusted *p* < 0.01

Arginine and proline metabolism was the next most important target of I3C in prostate tissues of Pten KO mice. This pathway is known to have profound effects on prostate cancer progression and tumor microenvironment [21, 22]. Based on metabolite data analysis, hydroxyproline was significantly upregulated by threefold by the genetic KO of Pten. However, I3C reversed this effect by nearly 1.6 fold. At the same time, metabolites like arginine and guanidinoacetate, were not substantially regulated by I3C in prostate tissues of Pten KO mice, although their levels were significantly changed with the suppression of Pten. (Figs. 5a and b).

#### I3C targets deregulated porphyrin metabolism in Pten KO mice prostate

**Fig. 6:**
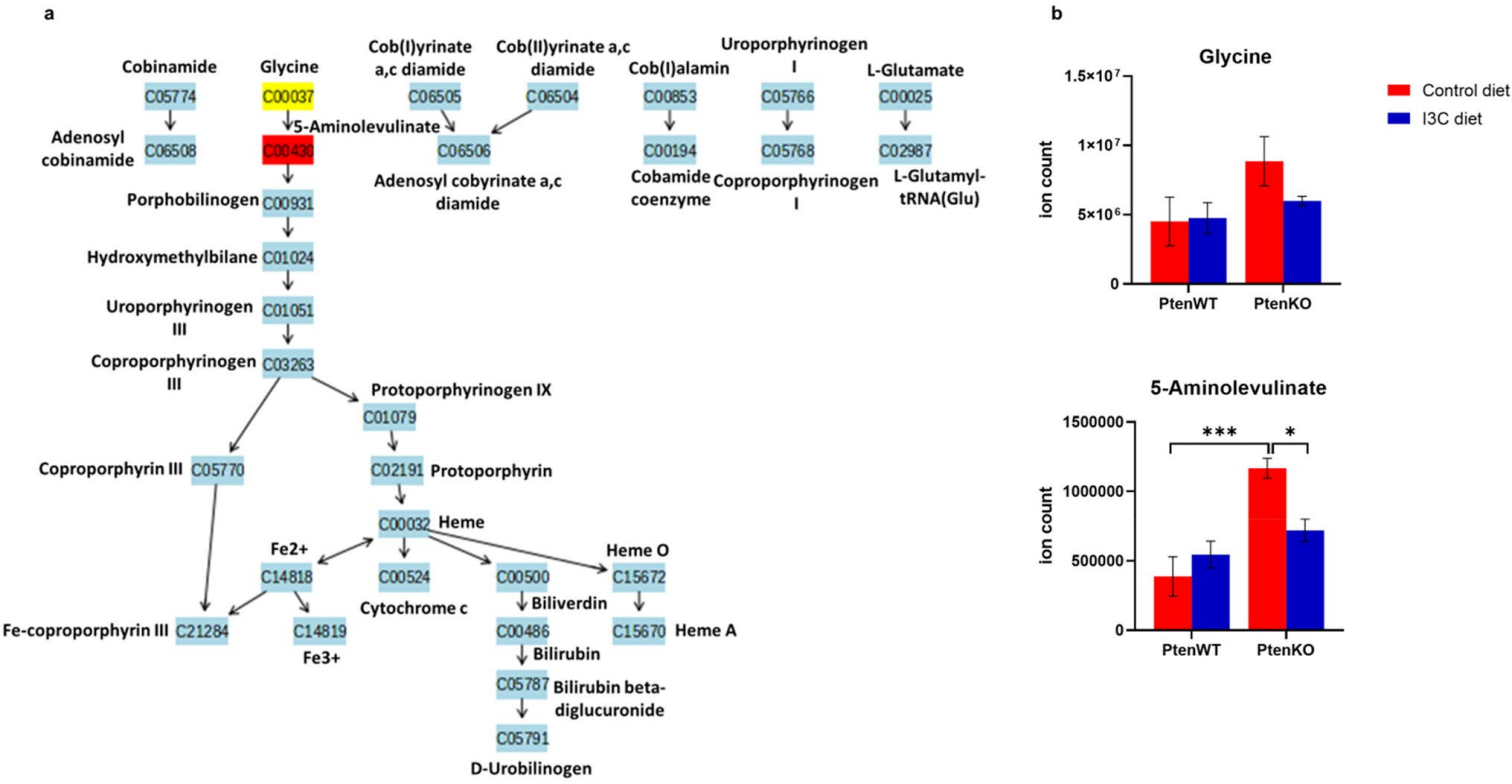
Regulation of Pten-dependent porphyrin metabolism by I3C in prostate of Pten KO mice. (**a**) Metabolite enrichment analysis based on I3C diet in Pten KO mice. Color coded compounds within the pathway: light blue represents those metabolites that are not in the data and are used as background for enrichment analysis; other colors (varying from yellow to red) represents the metabolites that are in the data with different levels of significance. (**b**) Expression of porphyrin metabolism metabolites in prostate tissues of different experimental groups is shown as mean ± SEM. *adjusted *p* < 0.05, **adjusted *p* < 0.01

It was interesting to note that I3C had the potential to target the disturbed heme biosynthesis pathway caused due to genetic KO of Pten. Heme biosynthesis pathway is often considered a cataplerotic pathway for the TCA cycle [23]. 5-Aminolevulinate, the first metabolite of this pathway and precursor of porphyrin metabolites exhibited threefold increase in the prostate of Pten KO mice. I3C diet was found to suppress the metabolite level by nearly 40% (Figs. 6a and b).

#### I3C influences Pten independent metabolic pathways in mouse prostate

In addition to directly regulating metabolic pathways dependent on Pten, I3C was found to significantly affect certain metabolites in prostate cancer mice model that were not necessarily regulated by Pten. Referencing metabolic pathway analysis described in Sect. " I3C regulates Pten dependent metabolic pathways in mice prostate"*,* we first compared figs. 3b and c and identified metabolic pathways regulated by I3C. We then selected Pten independent pathways by excluding those which were not modulated by Pten KO (Fig. 3a). Alternatively,

$${\varvec{P}}{\varvec{t}}{\varvec{e}}{\varvec{n}}\;\boldsymbol{ }{\varvec{i}}{\varvec{n}}{\varvec{d}}{\varvec{e}}{\varvec{p}}{\varvec{e}}{\varvec{n}}{\varvec{d}}{\varvec{e}}{\varvec{n}}{\varvec{t}}\;\boldsymbol{ }{\varvec{I}}3{\varvec{C}}\;\boldsymbol{ }{\varvec{t}}{\varvec{a}}{\varvec{r}}{\varvec{g}}{\varvec{e}}{\varvec{t}}{\varvec{e}}{\varvec{d}}\;\boldsymbol{ }{\varvec{m}}{\varvec{e}}{\varvec{t}}{\varvec{a}}{\varvec{b}}{\varvec{o}}{\varvec{l}}{\varvec{i}}{\varvec{c}}\;\boldsymbol{ }{\varvec{p}}{\varvec{a}}{\varvec{t}}{\varvec{h}}{\varvec{w}}{\varvec{a}}{\varvec{y}}{\varvec{s}}={\varvec{y}}\cap {\varvec{z}}-{\varvec{x}}$$

Venn diagram representing Pten independent metabolic pathways significantly targeted by I3C is shown in Fig. 3e. Based on this analysis, we determined citrate cycle and lipoic acid metabolism as the potential targets of I3C. The list of all identified metabolic pathways along with their statistical significance is provided in supplementary information.

#### I3C targets citrate cycle in Pten KO mice prostate

**Fig. 7:**
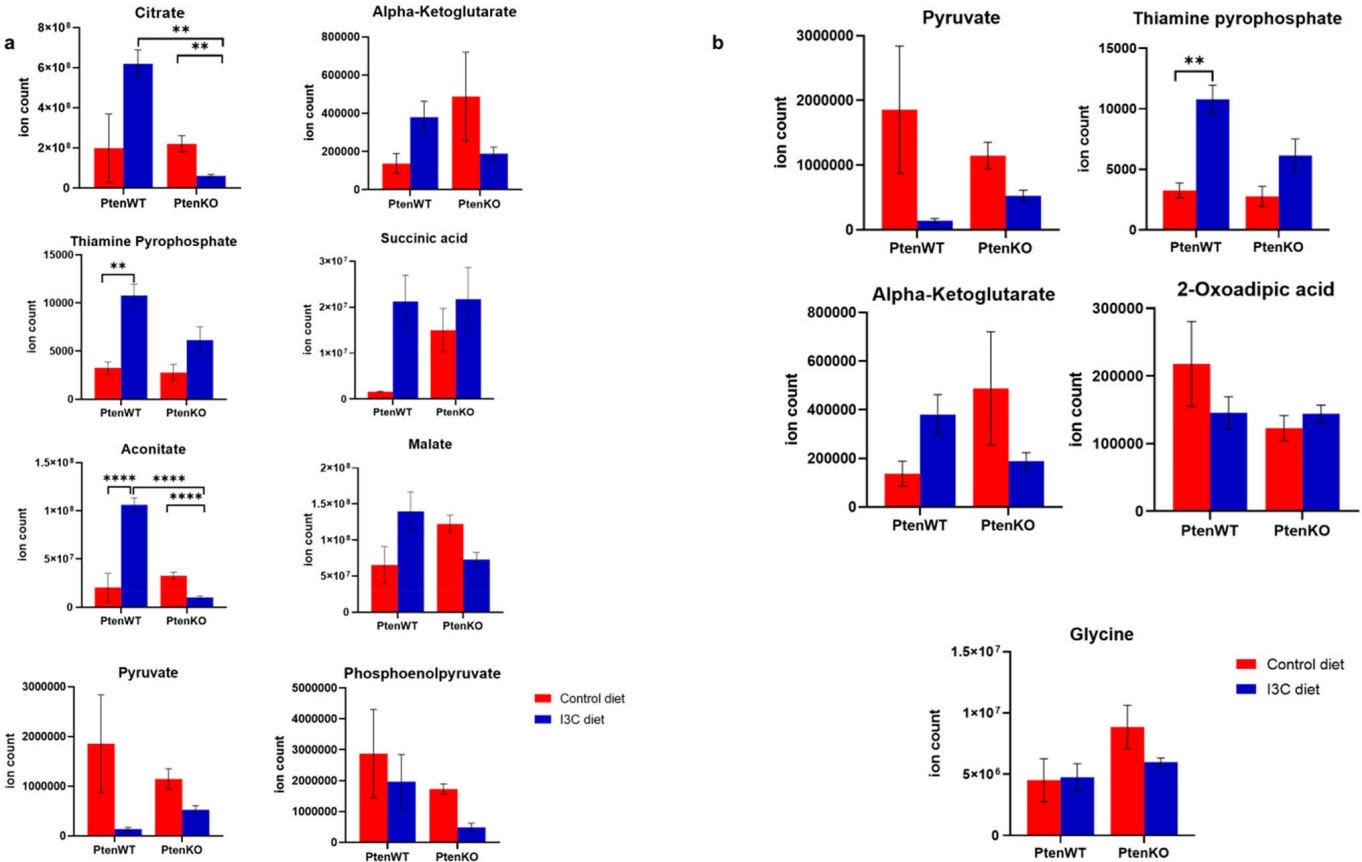
I3C targets Pten-independent metabolic pathways in prostate of Pten KO mice. (**a**) Regulation of metabolites of citrate cycle by I3C diet in Pten KO mice. (**b**) Regulation of metabolites of lipoic acid metabolism by I3C diet in Pten KO mice. Expression of metabolites is shown as mean ± SEM. *adjusted *p* < 0.05, **adjusted *p* < 0.01, ***adjusted *p* < 0.001, ****adjusted *p* < 0.00001

I3C significantly regulated mitochondrial energy metabolism by lowering the levels of citrate cycle metabolites in the prostate of Pten KO mice. We observed statistically significant decrease of 70.6% in citrateand 67.6% in aconitate **(**Fig. 7a**)**.

#### I3C targets lipoic acid metabolism in Pten KO mice prostate

Lipoic acid is an important cofactor of mitochondrial metabolism [24]. Among the identified metabolites in Pten KO mice prostate tissue, we did not observe statistically significant regulatory activity of I3C **(**Fig. 7b**)**. However, it is possible that unidentified metabolites may be metabolic targets of I3C.

## Discussion

Pten mediated cancer protection is known to promote oxidative phosphorylation and regulate metabolic signaling pathways including mitochondrial metabolism, lipid metabolism and glycogen synthesis [25, 26]. With deletion of Pten there is evidence of metabolic reprogramming in prostate cancer cells [27].To understand the anti-cancer potential of I3C, particularly at metabolomic level in prostate cancer based Pten KO mice model, we treated mice non-invasively with AIN-93 M diet containing 1% (w/w) I3C for 20 weeks. We designed a controlled experiment comprising Pten WT and Pten KO mice which were further subdivided into “control diet” and “I3C diet” groups based on diet fed to mice. Our major objective was to understand mechanism of action of I3C on prostate carcinogenesis based on metabolic alterations.

To test metabolic regulations in the prostate tissues of different groups of mice, we performed LC–MS based untargeted metabolomics. We identified the most regulated metabolic pathways potentially targeted by I3C in the chemoprevention of prostate cancer. Some of them were Pten-dependent and some others were Pten-independent. Pten-dependent pathways are expected to represent metabolic pathways based on interaction between I3C and Pten. These included pyrimidine metabolism, arginine and proline metabolism and porphyrin metabolism. Pyrimidine metabolism was found to be the most significantly regulated pathway caused by I3C diet. It is well understood that cancer cells reprogram metabolism to support vigorous cell growth by increasing pyrimidine de novo synthesis flux via steady supply of deoxyribonucleoside triphosphates (dNTPs) [28]. In addition, mammalian target of rapamycin complex 1 (mTORC1) cellular growth signaling is found to post translationally regulate de novo pyrimidine synthesis particularly the first three steps, during which N-carbamoyl aspartate (generated from glutamine) plays critical role in the formation of pyrimidine nucleotides [29]. Accumulating evidence suggest that abundance of N-carbamoyl aspartate is positively correlated with mTORC1 signaling [30]. I3C diet has shown great potential in inhibiting mTORC1 by suppressing N-carbamoyl aspartate in prostate cancer (Fig. 4b). Inhibition of dihydroorotate dehydrogenase (DHODH) which contributes to mitochondrial electron transport chain [31] and catalyzes conversion of dihydroorotate into orotate is regarded as a promising therapy to treat patients with Pten mutant cancers [32]. Interestingly, I3C diet significantly downregulated orotate levels in prostate tissues of Pten KO mice (Fig. 4b). Cytidine deaminases catalyze cytidine to uridine transitions and play a significant role in protecting cancer cells against deoxycytidine-based chemotherapies [33]. Inability of I3C diet to reverse the changes in uridine and cytidine levels in Pten KO based prostate cancer (Fig. 4b) unveils probable resistance of prostate cancer cells to I3C. Arginine and proline metabolism was determined as the next Pten dependent metabolomic target of I3C. With the deletion of Pten, mice prostate was starved of arginine but proline amount was stable. However, metabolite of proline, hydroxyproline was significantly increased by the loss of Pten and was decreased by I3C (Fig. 5b). An increased availability of hydroxyproline in solid tissue is associated with Hypoxia-inducible factor 1-alpha (HIF1-α) mediated cancer cell survival by promoting expression of matrix metalloproteinase and degradation of the extracellular matrix (ECM) [34]. It was interesting to note that I3C had the potential to target the elevated hydroxyproline in mice prostate carcinoma.. Alteration in porphyrin metabolism was also identified as Pten-dependent metabolic target of I3C. Although, external administration of 5-aminolevulinate is an approved therapeutic strategy in photodynamic cancer therapy [35], it is unclear what molecular mechanism substantially elevated this intermediate of heme biosynthesis in prostate of Pten KO mice. Furthermore, I3C was shown to downregulate 5-aminolevulinate with statistical significance (Fig. 6b).

Among the metabolic pathways which were not dependent on Pten, we determined citrate cycle and lipoic acid metabolism as potential metabolic targets of I3C. Prostate cancer cells demonstrate higher citric acid cycle activity relative to benign cells with net decrease in citrate secretion, unlike most cancer cells which resort to aerobic glycolysis [36, 37]. Surprisingly, I3C significantly downregulated several key metabolites of this pathway including citrateand aconitate (Fig. 7a) in prostate of Pten KO mice, suggesting pro-cancer effect of I3C. Regulation of citrate cycle by I3C is further expected to impact lipoic acid metabolism (Fig. 7b), an essential contributor to cell growth and mitochondrial metabolism [38].

I3C has been regarded as a chemopreventive agent due to its antioxidant activity, particularly via regulation of phase II drug metabolizing, antioxidant and apoptotic genes by Nrf2 [10, 14]. Nrf2 being a master regulator of cellular oxidative stress, its activation by I3C may have caused a survival mechanism to cope and thrive in early stages of Pten KO mediated prostate cancer tumor microenvironment leading to its progression which requires further investigation. Taken together, metabolic correlation between Pten and Nrf2 activator, I3C can be considered an emerging marker to diagnose and monitor prostate cancer.

## Conclusion

In summary, for the first time we identified some metabolic targets of I3C that describe its effects against Pten KO prostate cancer. We identified Pten-dependent (pyrimidine metabolism, arginine and proline metabolism and porphyrin metabolism) as well as Pten-independent (citrate cycle and lipoic acid metabolism) pathways targeted by I3C. Additional *in-vivo* studies are needed to determine the circumstances related to initial stages of carcinoma such as initiation, promotion or progression of prostate cancer development during which I3C might be suitable for anti-cancer effects including cancer prevention.

## Supplementary Information

Supplementary Material 1

Supplementary material 2

Supplementary Material 3

Supplementary Material 4

## Abbreviations

Akt: Ak strain transforming
dNTPs: Deoxyribonucleoside triphosphates
DHODH: Dihydroorotate dehydrogenase
DLP: Dorsal & lateral prostate
ECM: Extracellular matrix
HIF1-α: Hypoxia-inducible factor 1-alpha
I3C: Indole-3-carbinol
IACUC: Institutional Animal Care and Use Committee
KO: Knockout
LC-MS: Liquid-chromatography mass-spectrometry
mTORC1: Mammalian target of rapamycin complex 1
Nrf2: Nuclear factor (erythroid-derived 2)-like 2
NF-κB: Nuclear factor kappa B
Pten: Phosphatase and tensin homolog deleted on chromosome 10
PCR: Polymerase chain reaction
TCA: Tricarboxylic acid
VLP: Ventral & lateral prostate
WT: Wild-type
